# Power transformer online monitoring and state assessment method based on 2DCNN-BiLSTM multi-source feature fusion

**DOI:** 10.1371/journal.pone.0345949

**Published:** 2026-04-20

**Authors:** Wenbo Zhang, Li Liu, Chen Chen, Songze Wei, Ying Zhang, Zhiyuan Huo, Dongxing Li, Hong Yin

**Affiliations:** 1 State Grid Jibei Electric Power Co., Ltd, Luanping County Power Supply Branch Company, Chengde, China; 2 State Grid Chengde Power Supply Company, Chengde, China; 3 Chengde Haoyuan Electric Power Installation Group Co., Ltd, Chengde, China; Wuhan Institute of Technology, CHINA

## Abstract

As key power system equipment, real-time monitoring and accurate assessment of power transformers’ operating status are critical for grid safety. To address deficiencies in existing methods regarding multi-source heterogeneous data fusion, unstructured information processing, and small-sample fault recognition, this paper proposes a multi-source deep feature extraction and fusion method combining two-dimensional convolutional neural networks (2D-CNN) and bidirectional long short-term memory networks (BiLSTM) for online transformer status monitoring. A multi-channel sensor platform collects key variables (e.g., partial discharge acoustic signals, voltage, current, oil-dissolved gas concentration) to acquire and standardize spatial and temporal features. A dual-channel model is designed: 2D-CNN extracts spatial features from images, BiLSTM captures temporal dependencies, with an attention mechanism weighting and fusing these features, followed by a fully connected layer. A Softmax classifier with ensemble learning performs state discrimination to enhance stability and generalization. Experiments using real data from east China power grid Dongwu Station transformers for classifying five typical states show the method outperforms traditional and single-modal deep models in accuracy, achieving effective transformer status monitoring.

## 1 Introductions

Transformers play a crucial role in the operation of power systems, affecting the safety and reliability of power supply. Once a transformer fails, it can affect the safe operation of the power system and even lead to widespread power outages. With the continuous increase in demand for electricity and the steady growth of electricity load in current society, the reliable operation of transformers plays a crucial role in the power supply reliability of the power system. Therefore, achieving online monitoring and assessment of transformer status is of great significance for preventing power system failures and ensuring the reliability of power supply. Traditional transformer condition monitoring often relies on a single sensor to collect specific physical quantities, such as temperature, voltage, current, and dissolved gas content, and uses empirical formulas or thresholds to determine the condition of the transformer. However, there are problems such as low information utilization and low accuracy in judgment [1]. In recent years, with the rapid development of sensor technology and information technology, transformer online monitoring systems have gradually achieved synchronous collection of multi-source data [2], including heterogeneous data such as temperature sensing, electrical parameter measurement, dissolved gas analysis (DGA), infrared thermal imaging, and voiceprint signals [3,4]. The fusion of multi-source data provides technical support for in-depth exploration of the intrinsic correlation of transformer operation status. Deep learning has demonstrated outstanding feature learning capabilities in fields such as image recognition, speech processing, and time-series data analysis.

At present, scholars have made significant progress in the field of transformer condition assessment in multiple aspects. In terms of online monitoring technology, Manoj et al. [5] combined the Analytic Hierarchy Process with Grey Relational Analysis to construct a fuzzy assessment model for transformer health status, effectively addressing the uncertainty issues in the assessment process. Schiewaldt et al. [6] evaluated the application effect of UHF frequency range in transformer fault classification, providing a theoretical basis for optimizing partial discharge detection technology. Liu et al. [7] combined Raman spectroscopy technology and optimized support vector machines to establish an assessment method for the aging state of transformer oil paper insulation, achieving accurate judgment of the degree of insulation system degradation. In terms of deep learning applications, researchers have introduced advanced neural network technology into the field of transformer state monitoring. Wang et al. [8] proposed interactive transformers and CNN networks for fusion classification of hyperspectral and LiDAR data, demonstrating the advantages of deep learning in multimodal data processing. Lu et al. [9] achieved degradation trend measurement of aircraft engine feature hierarchy fusion based on parameter adaptive VMD method and improved transformer model. Liu et al. [10] proposed a digital twin assembly model based on visual transformers for multi-source mutation fusion, providing a new approach for complex system state monitoring. Shang et al. [11] combined SGMD approximate entropy feature extraction technology with optimized BiLSTM network to construct a partial discharge fault diagnosis model for transformers, significantly improving the accuracy of fault identification. Liu et al. [12] proposed a dual transformer BiLSTM network, which improves the robustness of speech features through a dual transformer structure and provides a new method for speech emotion recognition. Wang et al. [13] used LSTM model for dynamic early fault prediction of power transformers, achieving early warning of faults. Guangliang et al. [14] demonstrated the advantages of temporal modeling by using transformers and stacked Bi LSTM for multi-channel and multi-step spectrum prediction. Pentsos et al. [15] proposed a hybrid LSTM transformer model for power load forecasting and verified the effectiveness of the hybrid architecture. Mitiche et al. [16] used LSTM autoencoders for data-driven anomaly detection in high-voltage transformer bushings, providing a new technological means for equipment health monitoring.

In the research of transformer state assessment methods, scholars have promoted technological innovation from multiple perspectives. The assessment method based on subsystem measurement improves the accuracy and interpretability of assessment through data and knowledge fusion [17]. The fuzzy assessment model combining Analytic Hierarchy Process and Grey Relational Analysis effectively addresses uncertainty issues in assessment [18,19]. The mixed failure mode analysis framework has achieved the quantification of component risk priority in a hesitant fuzzy environment [20], providing a scientific basis for maintenance strategies. However, existing methods still have shortcomings in multi-source heterogeneous data fusion, unstructured information processing, and small sample fault recognition, and cannot effectively utilize multi-source heterogeneous data for transformer operation status assessment, with low accuracy.

In response to the above issues, this paper proposes a multi-source feature fusion transformer online monitoring and state assessment method based on dimensional convolutional neural networks (2D-CNN) and bidirectional long short-term memory networks (BiLSTM). In Section 2, a multi-sensor data acquisition system was constructed to provide synchronous acquisition and processing methods for multi-source data. In Section 3, a feature extraction model for 2DCNN-BiLSTM was designed, which can extract spatial and temporal features. In Section 4, a transformer state assessment model is proposed, which utilizes a fully connected layer combined with attention mechanism to achieve efficient fusion of multi-source features, effectively solving the problem of small samples, and using Softmax layer to complete classification tasks. In Section 5, by obtaining operational data of 1000kV transformers and comparing and analyzing the assessment effect of transformer health status using various methods, the effectiveness and accuracy of the methods proposed in the article were verified. Finally, concluding remarks are mentioned in Section 6.

## 2 Multi-sensor state monitoring system

### 2.1 Transformer structure and common types of faults

**Fig 1:**
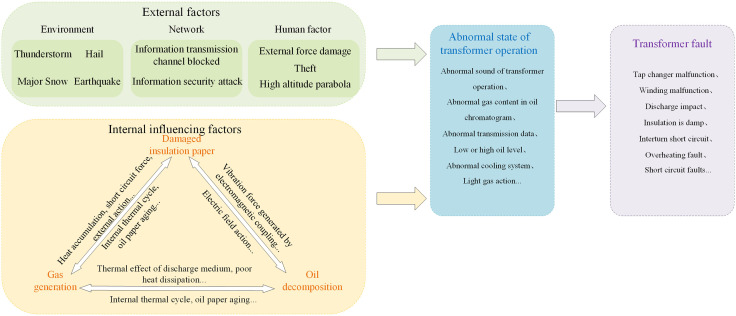
Factors affecting transformer faults.

As an important equipment in the power system, transformers are mainly composed of iron cores, windings, insulation systems, oil tanks, cooling systems, tap changers, and bushings. Transformers may malfunction during operation due to their own or environmental factors (as shown in Fig 1), leading to system abnormalities or failures.

Transformers face various environmental and operational changes during operation, and are prone to typical types of faults including winding failures, insulation degradation, abnormal iron cores, oil degradation, and cooling system failures. Winding faults are usually caused by overload, electrical shock, or insulation failure, manifested as local overheating, inter turn short circuits, and an increase in combustible gas concentration in the oil. Insulation systems are prone to performance degradation under long-term thermal stress and humid environments, leading to a decrease in insulation resistance and an increase in dielectric loss factor, which can further cause partial discharge and even breakdown accidents. Iron core faults are mostly caused by structural looseness or abnormal grounding, resulting in local magnetic loss and increased heat generation, as well as significant noise. The quality change of insulating oil directly affects the cooling and electrical insulation performance of the system, and its deterioration is often accompanied by an increase in acid value, a decrease in breakdown voltage, and abnormal gas composition. Cooling system failures such as fan damage, oil pump failure, or blocked heat dissipation channels will seriously weaken the heat exchange capacity, causing an increase in oil temperature and system overheating.

Due to the fact that the above-mentioned types of faults often exhibit the characteristics of multi-source heterogeneity, it is difficult to distinguish them early, and a single physical quantity monitoring method is difficult to comprehensively reflect the operating status of the equipment. Therefore, establishing a state monitoring system based on multi-source sensing and integrating deep features is of great significance for improving the safety of transformer operation and the reliability of power system operation.

### 2.2 Architecture and composition of monitoring system

**Fig 2:**
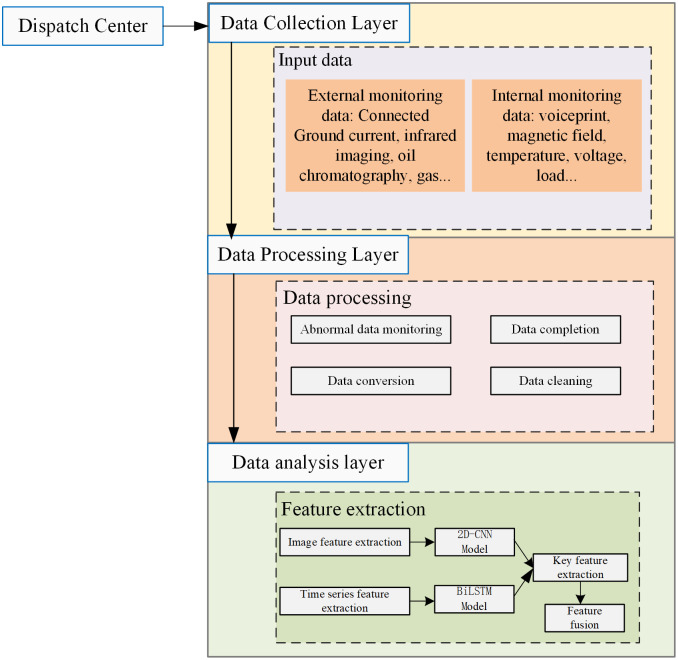
Architecture diagram of state monitoring system.

According to the functions of each module in the transformer system, this article divides the transformer status monitoring system into a three-layer monitoring architecture of “data acquisition layer data processing layer data analysis layer,” as shown in Fig 2.

Communication between layers is achieved through standardized interfaces, forming a complete information loop from low-level data collection to high-level intelligent analysis. The data collection layer transmits the operational status data of different components in the transformer monitored by multiple types of sensors to the data processing layer; The real-time preprocessing of multi-source data by the data processing layer significantly reduces data transmission pressure and improves system robustness; Finally, the data analysis layer used a hybrid architecture model of 2D-CNN and BiLSTM to extract and fuse key features of multimodal features, achieving accurate recognition and prediction of transformer states.

## 3 Feature extraction model based on 2D-CNN and Bi LSTM

This article proposes a deep hybrid feature extraction framework that combines two-dimensional convolutional neural networks (2D-CNN) and bidirectional long short-term memory networks (BiLSTM), and constructs an end-to-end multimodal state assessment model by combining fully connected layers and attention mechanisms.

### 3.1 Design of 2D-CNN spatial feature extraction module

In the multi-source transformer monitoring system, the image data, including infrared thermal image and voiceprint spectrum, have typical two-dimensional spatial structure characteristics, which is suitable for using convolutional neural network to extract high-order representation features in local regions. In order to make full use of the spatial information of image data, this part constructs an image feature extraction module based on two-dimensional convolutional network (2D-CNN). The external diagram reflects the temperature distribution of windings, bushings, oil tanks and other parts, which is an important basis for identifying thermal faults. By processing the mechanical vibration signal with short-time Fourier transform (STFT) and Mel filter banks, the voiceprint pattern can obtain the time-frequency distribution characteristic map, which has the time-frequency joint characteristics and is suitable for depicting the peak of partial discharge or abnormal vibration mode.

Let the discrete acoustic signal be *x*[*n*], the window function be *w*[*n*], the window length be *N*, the sliding step be *S*. The STFT expression is as follows:


$$X_{a}(m,k)=\sum_{n=\text{0}}^{N-\text{1}}x[n+mS]•w[n]•e^{-j\frac{\text{2}\pi kn}{N}},\text{0}\leq k<N$$ (1)

Where *m* denotes the index number of the current frame, *X*_*a*_(*m*, *k*) represents the STFT complex result of the *m*-th frame at the *k*-th frequency, and *j* is the imaginary unit, where *j*^2^ = −1.

Calculate the square of the frequency spectrum modulus and take the logarithm to construct a logarithmic power spectrum, as shown below:


$$LogSpec\left(m,k\right)=log({\left|X_{a}\left(m,k\right)\right|}^{\text{2}}+\varepsilon )$$ (2)

Among them, *LogSpec*(*m*, *k*) is the logarithmic power spectrum value of the *m*-th frame at the *k*-th frequency, and *ε* is a small quantity.

Subsequently, Mel filter banks were introduced to weight each frame of the spectrogram, obtaining a spectral response that is closer to human auditory perception. The response function of the *m*-th filter at frequency *k* is:


$$H_{m}\left(k\right)=\left\{ \begin{array}{c} @c0,\;\;k<f(m-1)\\ \frac{2(k-f(m-1))}{\left(f(m)-f(m-1)\right)\left(f(m+1)-f(m-1)\right)},f(m-1)\leq k<f\left(m\right)\mathrm{\backslash vspace}1mm\\ \frac{2(f(m+1)-k)}{\left(f(m+1)-f(m)\right)\left(f(m+1)-f(m-1)\right)},f(m-1)lek<f(m+1)\mathrm{\backslash vspace}1mm\\ 0,\;\;k>f(m+1) \end{array}\right.$$ (3)

Where *H*_*m*_(*k*) is the response value of the *m*-th filter at the *k*-th frequency, and *f*(*m*) is the center frequency corresponding to the *m*-th filter.

The final Mel spectrogram is obtained by weighting and accumulating the filters of each frequency band, as shown below:


$$Melspec\left(t,m\right)=\sum_{k=0}^{k-1}{\left|X_{a}\left(t,k\right)\right|}^{\text{2}}\cdot H_{m}\left(k\right)$$ (4)

Where *Melspec*(*t*, *m*) is the output result of the *m*-th Mel filter in the *t*-th frame, and *H*_*m*_(*k*) is the weight coefficient of the *m*-th filter at frequency *k*.

The obtained two-dimensional image is the voiceprint image, which can be combined with the infrared image as an image input and sent to the 2D-CNN module for spatial feature extraction. All images are uniformly cropped to size H*W before input and normalized. The 2D-CNN network structure adopts a combination module of “convolutional layer activation function pooling layer” to extract local spatial features of the image layer by layer. The core convolution calculation is expressed as:


$$\overset{\left(l\right)}{\underset{k}{F}}(i,j)=\sigma \left(\sum_{c=1}^{C}\sum_{u=1}^{K}\sum_{v=1}^{K}\overset{\left(l\right)}{\underset{k,c}{W}}(u,v)⬝I_{c}(i+u,j+v)+\overset{\left(l\right)}{\underset{k}{b}}\right)$$ (5)

Where $I\in {\mathbb{R}}^{H\times W\times C}$ is the input image tensor, $\overset{\left(l\right)}{\underset{k,c}{W}}\in {\mathbb{R}}^{K\times K}$ represents the weight of the *k*-th convolution kernel in the *l*-th layer on the *c*-th channel, $\overset{\left(l\right)}{\underset{k}{b}}$ is the bias term, *K* is the size of the convolution kerne*l*, (*i*,*j*) is the image position coordinate, and *σ*(·) is the nonlinear activation function.

After convolution, pooling operation is performed to compress the spatial dimension, reduce computational complexity, and enhance the translation invariance of features. Finally, it is flattened into a one-dimensional vector $f_{img}\in R^{d_{\text{1}}}$, which serves as the spatial feature representation of the image channel for subsequent multimodal fusion modules. In addition, to improve the stability and generalization ability of the model training, a Batch Normalization layer is added after the convolutional layer to standardize the activation output, and Dropout is introduced in some layers to suppress overfitting.

### 3.2 BiLSTM time series modeling module

#### 3.2.1 Principle of BiLSTM network structure.

In the intelligent sensing system for transformer status, time-series monitoring data such as winding temperature, voltage and current waveforms, and changes in gas concentration in oil contain dynamic evolution information of equipment operating status, with significant temporal correlation and long-term dependence structure. However, traditional feedforward neural networks are unable to effectively model such dynamic relationships, making it difficult to capture local abnormal fluctuations or non-stationary behavior. To this end, this article introduces a bidirectional long short-term memory network (BiLSTM) to deeply explore the evolution patterns and fault precursor features in temporal signals.

#### 3.2.2 Temporal feature extraction mechanism.

Let the standardized time-series sample sequence be:


$$X=(X_{\text{1}},X_{\text{2}},...,X_{T}),X_{t}\in {\mathbb{R}}^{d}$$ (6)

Where *T* is the length of the time series, *X*_*t*_ is the observation vector of the *t*-th time step, and the dimension is *d*.

The BiLSTM network performs bidirectional encoding on the processed time series, where the output of step *t* is represented as:


$$h_{t}=[\vec{h_{t}};\overset{\leftarrow }{h_{t}}],h_{t}\in {\mathbb{R}}^{2h}$$ (7)

Where $\vec{h_{t}}$ and $\overset{\leftarrow }{h_{t}}$ respectively represent the hidden state outputs of forward and backward LSTM, [·;·] represents the concatenation operation, and *h*_*t*_ is the hidden unit dimension in each direction.


$$\langle \begin{array}{c} i_{t}=\sigma (W_{i}X_{\text{t}}+U_{i}h_{t-1}+b_{i})\left(\text{input gate}\right)\\ f_{t}=\sigma (W_{f}X_{\text{t}}+U_{f}h_{t-1}+b_{f})\left(\text{forget gate}\right)\\ o_{t}=\sigma (W_{o}X_{\text{t}}+U_{o}h_{t-1}+b_{o})\left(\text{output gate}\right)\\ {\tilde{c}}_{t}=tanh(W_{c}X_{\text{t}}+U_{c}h_{t-1}+b_{c})\left(\text{candidate memory}\right)\\ c_{t}=f_{t}⊙c_{t-1}+i_{t}⊙{\tilde{c}}_{t}\left(\text{memory update}\right)\\ h_{t}=o_{t}⊙tanh\left(c_{t}\right)\left(\text{hidden state output}\right) \end{array}$$ (8)

Where $W_{*},U_{*}$ and $b_{*}$ are trainable parameters, tanh is the hyperbolic tangent function, and the symbol ⊙ represents element wise multiplication.

### 3.3 Feature fusion and attention mechanism design

By using global average pooling to reduce feature dimensions and enhance the modeling ability of temporal global trends, the bidirectional hidden state outputs of all time steps are integrated to obtain the final temporal channel features, which are represented as follows:


$$f_{seq}=\frac{\text{1}}{T}\sum_{t=1}^{T}h_{t},f_{seq}\in {\mathbb{R}}^{2h}$$ (9)

Among them, each physical quantity is described in equations (6) and (7).

The evolution trend and dynamic dependency characteristics of the monitoring signal are effectively encoded through vector *f*_*seq*_, serving as a global embedding representation of the temporal channel, providing high-quality input for subsequent multimodal feature fusion and state recognition. After obtaining the image feature vector and temporal feature vector, it is necessary to perform fusion modeling to achieve multi-source information collaborative perception. This article adopts a feature level fusion strategy and uses concatenation operations to:


$$f_{u}=\phi (W_{f}⬝[f_{img};f_{seq}]+b_{f}\text{)}$$ (10)

Where *Ф*(·) is the ReLU activation function, and *W*_*f*_ and *b*_*f*_ are the fusion layer weights.

To enhance the responsiveness of the model to key features, this paper introduces channel attention mechanism in the feature fusion stage and designs a fusion based attention weighting mechanism combined with multi-source information structure. Specifically, assuming that the spatial feature vector extracted by 2D-CNN is $F_{\text{cnn}}\in {\mathbb{R}}^{n\times d_{\text{1}}}$ and the temporal feature vector extracted by BiLSTM is $F_{\text{lstm}}\in {\mathbb{R}}^{n\times d_{\text{2}}}$, the two are first concatenated to obtain a fused feature matrix:


$$F=[F_{cnn};F_{lstm}]\in {\mathbb{R}}^{n\times (d_{\text{1}}+d_{\text{2}})}$$ (11)

Subsequently, a set of trainable channel attention weights $\alpha \in {\mathbb{R}}^{1\times (d_{\text{1}}+d_{\text{2}})}$ is defined and normalized using the Softmax function to obtain the weighting coefficients for each fusion channel:


$$\alpha_{i}=\frac{e^{w_{i}}}{\overset{d_{\text{1}}+d_{\text{2}}}{\underset{j=1}{\sum }}e^{w_{j}}},i=1,2,...,d_{\text{1}}+d_{\text{2}}$$ (12)

The weighted feature representation after final fusion is:


$$F_{att}=\alpha \cdot F$$ (13)

This channel attention mechanism can adaptively learn the importance of various feature channels during the training process, highlighting key dimensions and suppressing redundant features, thereby improving the classification accuracy and generalization ability of the model.

Further introduce multi head attention mechanism to extend the channel weighting process mentioned above. Each attention head learns different feature subspaces and independently calculates weighting coefficients, and finally obtains richer semantic expressions through concatenation operations. This strategy can effectively capture the interactive relationships between multi-scale and heterogeneous data, enhancing the expressive power of feature fusion and the interpretability of the model. The fused feature vectors will be input into a fully connected layer for nonlinear mapping, and ultimately implemented for state recognition through a Softmax classifier.

### 3.4 Design of state classifier and fault recognition module

The multimodal fusion features are fed into the fully connected layer and mapped to the class probability space output through the Softmax activation function:


$${\hat{y}}_{k}=\frac{exp(\overset{T}{\underset{\text{k}}{W}}f_{attn}+b_{k})}{\overset{K}{\underset{j=1}{\sum }}exp(\overset{T}{\underset{\text{j}}{W}}f_{attn}+b_{j})},k==1,2,...,K$$ (14)

Finally, the cross entropy loss function model is used to optimize the mapping from feature vectors to fault categories, achieving automatic recognition of operational status.


$$L=-\sum_{k=1}^{K}y_{k}log\left({\hat{y}}_{k}\right)$$ (15)

Formulas (14) and (15) represent the output layer and loss calculation of the classification model. Firstly, the output of the fully connected layer is converted into probability distributions for each category using the Softmax activation function. Then, the cross entropy loss function is used to measure the difference between the predicted probability and the true label. By minimizing this loss, the model parameters are optimized to achieve accurate classification prediction of transformer states.

### 3.5 Integrated algorithm

The integrated learning module uses the soft voting strategy to weighted average the classification output probabilities under multiple training rounds to avoid the impact of single prediction fluctuation on classification decision. The voting weight of each sub model is dynamically allocated based on its accuracy in the verification set, so as to improve the stability and robustness of the final judgment. Through three models with different initialization parameters, the category probability vectors of their respective outputs are weighted average:


$$P_{final}=\sum_{i=1}^{M}\omega_{i}⬝P_{i}$$ (16)

Where *ω*_*i*_ is the model weight. The final prediction category is determined by the label corresponding to the maximum probability, which is shown as follows:


$$\hat{y}=arg\;max\left(P_{final}\right)$$ (17)

The integrated algorithm can effectively reduce the bias and over fitting of individual models and improve the overall prediction performance of the model.

## 4 Transformer condition assessment model based on integrated algorithm

Based on the successful extraction of multimodal spatial and temporal features by 2D-CNN and BiLSTM modules, this chapter designs a state assessment model of multi-head attention mechanism and integrated learning strategies. Through the construction of end-to-end training process, the task adaptability loss function and integrated optimization mechanism are introduced to realize the efficient classification and intelligent perception of transformer operation state under multi-source input.

### 4.1 Model training process

**Fig 3:**
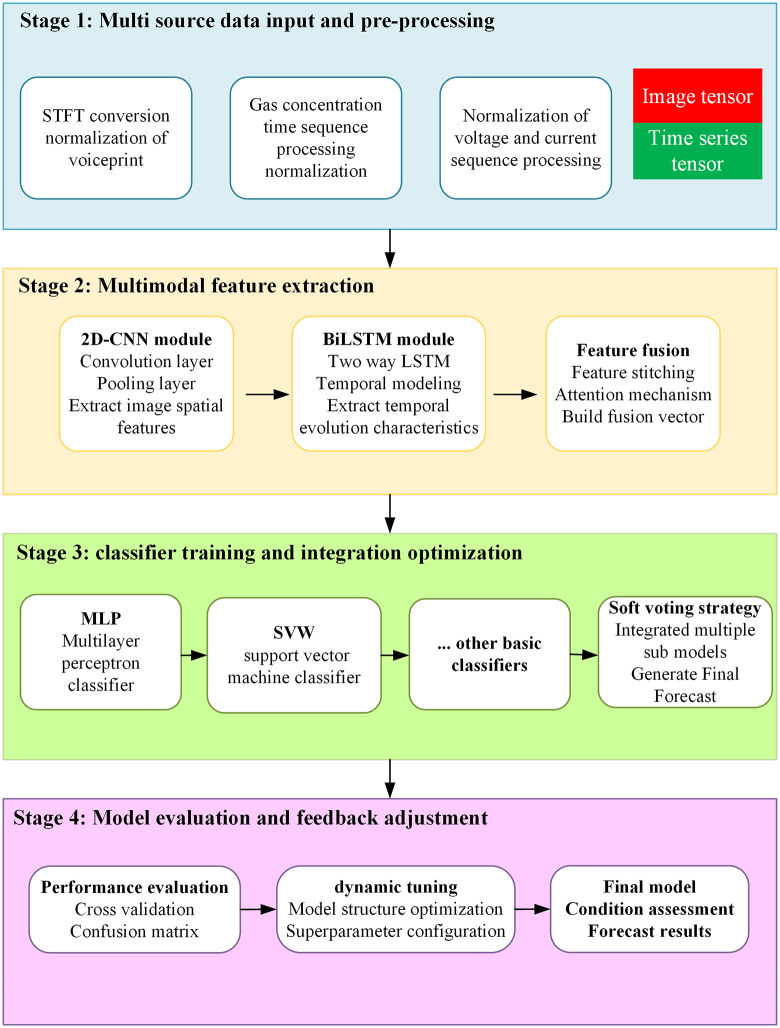
Flow chart of model training.

The overall training process of the state evaluation model proposed in this paper is shown in Fig 3. It mainly includes the following four stages: ①multi source data input and preprocessing: obtain the information such as voiceprint map, gas concentration, voltage and current during the operation of the transformer, and construct the standard input quantity of image type and time sequence type through normalization, clipping, STFT conversion and other operations respectively. ②multimodal feature extraction: 2D-CNN module is used to extract image spatial features, BiLSTM module is used to extract temporal evolution features, and the fusion vector is constructed through feature stitching and attention mechanism. ③classifier training and integration optimization: input the fusion vector into multiple basic classifiers, construct multiple sub model outputs, and use the soft voting strategy to generate the final prediction label. ④model assessment and feedback adjustment: indicators such as cross validation and confusion matrix are introduced to assess the performance of the model, and the model structure and super parameter configuration are dynamically adjusted accordingly.

### 4.2 Loss function and algorithm optimization

#### 4.2.1 Definition of classification loss function.

The essence of the state recognition task is a multi classification problem. The cross entropy loss function is defined as follows:


$$L_{CE}=-\sum_{Z=1}^{Z}y_{Z}log\left({\hat{y}}_{Z}\right)$$ (18)

Where *Z* is the number of state categories, *y*_*Z*_∈{0,1} is the unique hot code of the actual tag, and ${\hat{y}}_{Z}$ is the prediction probability of the model for the *k*-th category.

To enhance the sensitivity to minority classes, the category weighting strategy is introduced to obtain the weighted cross entropy loss, which is expressed as follows:


$$L_{WCE}=-\sum_{Z=1}^{Z}w_{Z}y_{Z}log\left({\hat{y}}_{Z}\right)$$ (19)

Where *w*_*Z*_ is the weight of the *Z*-th sample, which is set by the reciprocal of the sample frequency.

#### 4.2.2 Optimizer and regularization strategy.

In order to improve the convergence efficiency and generalization ability of the model, Adam algorithm is selected in the training process, combined with first-order momentum and second-order correction to improve the gradient update efficiency. Add dropout after the full connection layer to prevent over fitting, and add L2 regular items, as shown below:


$$L_{total}=L_{WCE}+\lambda \overset{\text{2}}{\underset{\text{2}}{\|\theta \|}}$$ (20)

Where *θ* is the model parameter set and *λ* is the regularization strength superparameter.

In order to further improve the robustness and generalization performance of the model, this paper uses the soft voting method to classify the final state results of the transformer, and the final classification results take the category corresponding to the maximum probability.

### 4.3 Overall assessment framework

**Fig 4:**
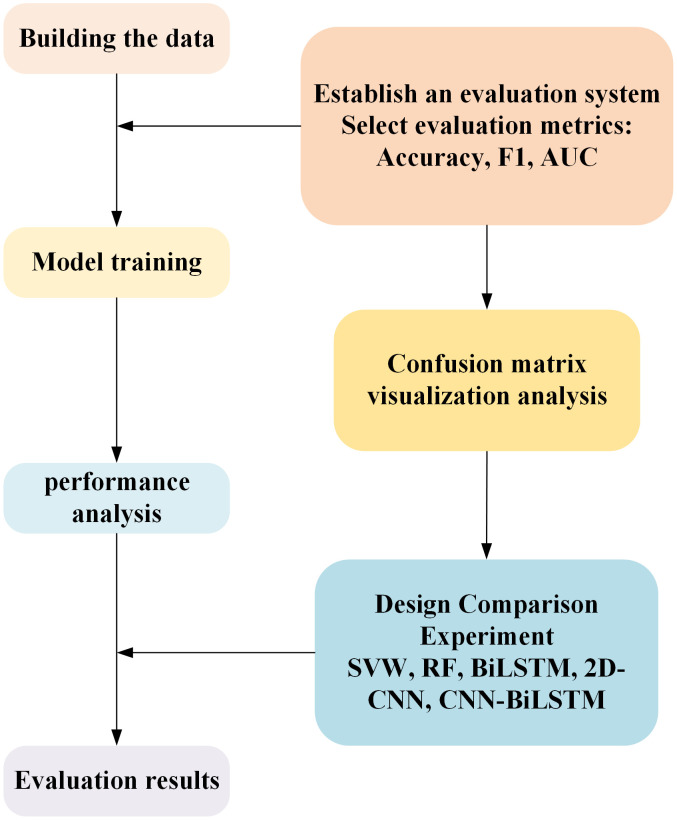
Assessment process.

In order to comprehensively verify the effectiveness of the proposed 2D-CNN-BiLSTM multi-source feature fusion method in transformer condition assessment and ensure the scientificity and credibility of the experimental results, the assessment process constructed in this paper is shown in Fig 4.

## 5 Example analysis

### 5.1 Experimental dataset and sample construction

**Fig 5:**
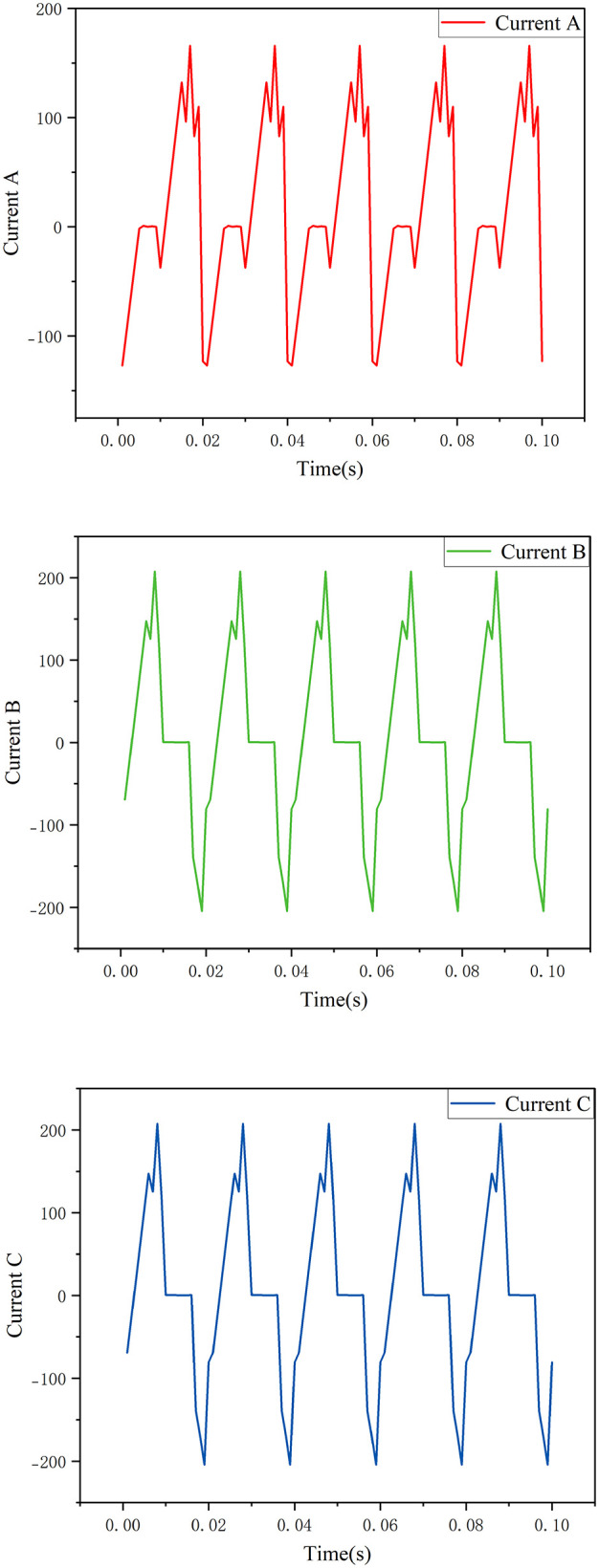
Typical current timing curve.

**Fig 6:**
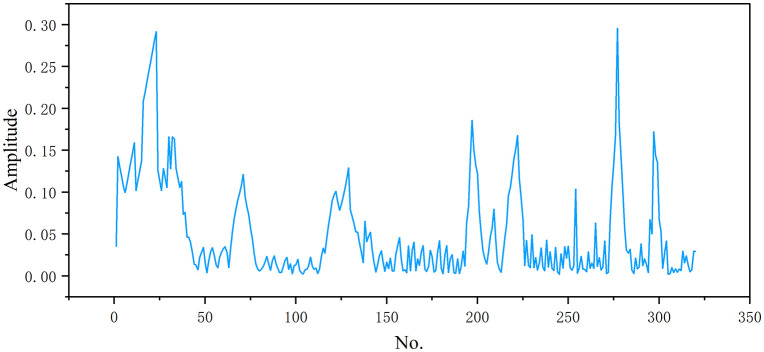
Vibration signal.

In order to verify the effectiveness of the multi-source deep fusion state assessment model proposed in this paper, this section selects a 1000 kV UHV main transformer in Dongwu substation of East China power grid as the test object to carry out multi-source online monitoring data acquisition and modeling experiments. The monitoring data collected include seven typical combustible gas concentrations, voltage and current signals and voiceprint data, such as dissolved gas in oil (DGA) data (H_2_, CH_4_, C_2_H_2_, C_2_H_4_, C_2_H₆, CO, CO_2_). In the related research of transformer condition monitoring, the literature provides a theoretical basis for DGA data analysis and feature extraction of current data [21–24]. Among them, Fig 5 shows the time sequence change curve of typical three-phase currents of transformers a, B and C. Fig 6 shows the amplitude distribution of vibration signal, reflecting the vibration intensity law during equipment operation.

A total of 3240 valid samples were collected in the experiment, and the category distribution was as follows: 1458 (45%) were in normal operation, 648 (20%) were in winding overheating, 486 (15%) were in insulation deterioration, 389 (12%) were in abnormal iron core, and 259 (8%) were in cooling system failure. The obvious abnormal data points are eliminated by using the 3σ criterion, and the missing values are completed by linear interpolation. Z-score standardization is carried out for each type of feature to ensure the comparability of different dimensional features. The sliding window technology is adopted. The window length is set to 64 time steps and the sliding step length is set to 8 to ensure appropriate overlap between samples to increase data diversity. Based on manual patrol marking and expert experience judgment, the original samples are divided into five typical operation status categories in Table 1. In order to deal with the problem of category imbalance, hierarchical sampling strategy is adopted to ensure that the proportion of each category in the training, verification and test sets is consistent. For a small number of samples, the time series data perturbation method is used to enhance, and the inverse proportional weight is introduced into the loss function. The sample set is divided into training set, verification set and test set according to the proportion of 70%: 15%: 15%. The classification of five typical operation status categories is shown in Table 1.

**Table 1:** Five typical operation status categories.

Categories	Status name	Explains	Proportion
C_1_	Normal operation	The equipment operation parameters are normal without obvious abnormal characteristics	45%
C_2_	Winding overheating	The current fluctuates violently, the heat load exceeds the limit, and the oil temperature rises abnormally	20%
C_3_	Insulation deterioration	The concentration of CO, C_2_H_2_ and other gases in DGA increases and the electrical strength decreases	15%
C_4_	Abnormal iron core	Abnormal enhancement in high frequency region of voiceprint spectrum, accompanied by noise and local magnetic saturation	12%
C_5_	Cooling system fault	The oil temperature rises rapidly, the oil level drops, and the air cooling unit fails or is blocked	8%

### 5.2 Model structure and training parameter setting

**Fig 7:**
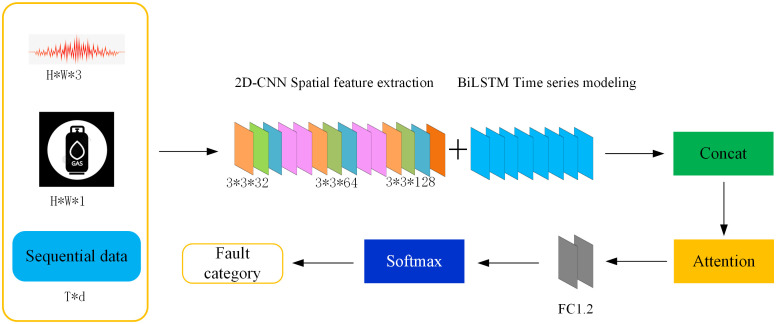
2D.CNN-BiLSTM neural network model.

The fusion model proposed in this paper is based on two-dimensional convolutional neural network (2D-CNN) and bidirectional long-short term memory network (BiLSTM) to realize the depth extraction and fusion discrimination of image and time series features. The network structure is shown in Fig 7. The two channel output feature vectors are weighted and fused through the attention mechanism to highlight the key channels, and feature fusion is performed through the full connection layer output. Finally, the final fault classification is completed using the softmax classifier. The cross entropy loss function is used and added in the training process. The optimizer selects Adam, the initial learning rate is 0.0001, the batch size is 32, and the number of training rounds is 100, so that the model training can avoid oscillation and ensure complete convergence.

**Fig 8:**
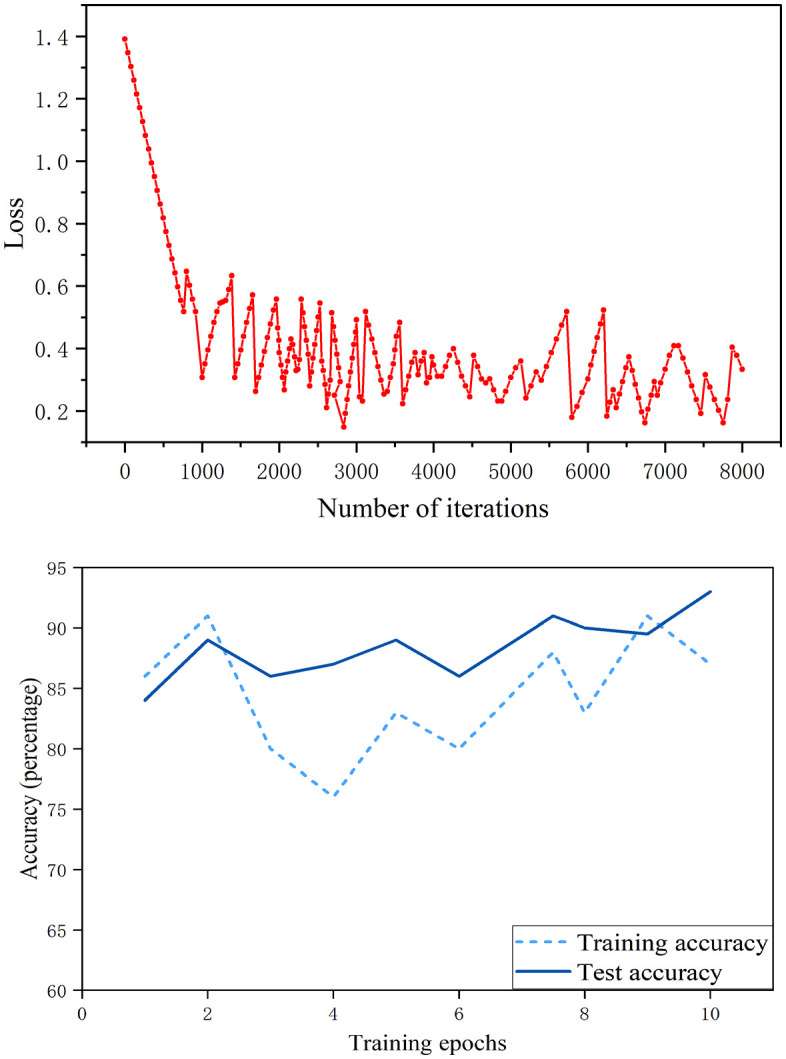
Model performance. (a) Loss function. (b) Training accuracy and test accuracy.

The performance of the proposed fusion model is analyzed, as shown in Fig 8. It can be seen from Fig 8 (a) that with the increase of iteration times, the loss value gradually decreases and tends to be stable, and the convergence effect of the model is good. According to the accuracy curve in Fig 8 (b), the accuracy of the training set and the test set increased synchronously, and finally stabilized at a high level without obvious over fitting.

### 5.3 Analysis of assessment results

In terms of verifying the effectiveness and superiority of the proposed multi-source feature fusion modeling method, this paper makes a systematic comparative analysis with the current mainstream transformer condition assessment methods [21–24]. The selected comparison models include: traditional machine learning algorithms support vector machine (SVM) and random forest (RF), which are highly representative in simple structure and small sample learning. In the deep learning model, bilstm structure and 2d-cnn structure are selected respectively to verify the independent performance of temporal modeling and spatial modeling ability. In addition, in order to evaluate the impact of attention mechanism on fusion performance, CNN bilstm combined model without attention mechanism is set as the comparison benchmark. Finally, the multi-source depth feature fusion model, which integrates 2d-cnn and bilstm and introduces attention mechanism, is used for comprehensive comparison. By unifying the data set, model configuration and assessment index, the performance of each model in fault identification accuracy, generalization ability, robustness and calculation efficiency is compared, so as to comprehensively evaluate the application advantages of this method in the task of transformer condition monitoring and assessment.

**Fig 9:**
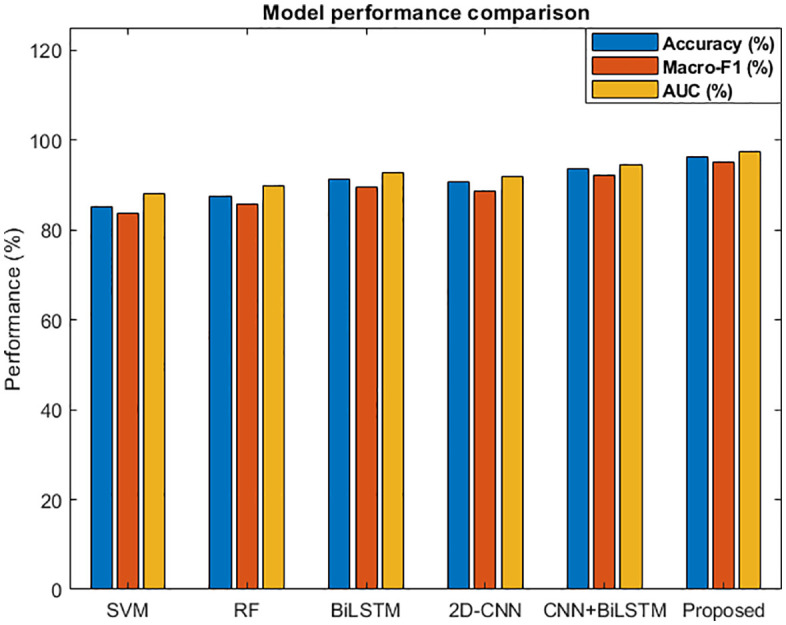
Comparison of Model Performance.

**Fig 10:**
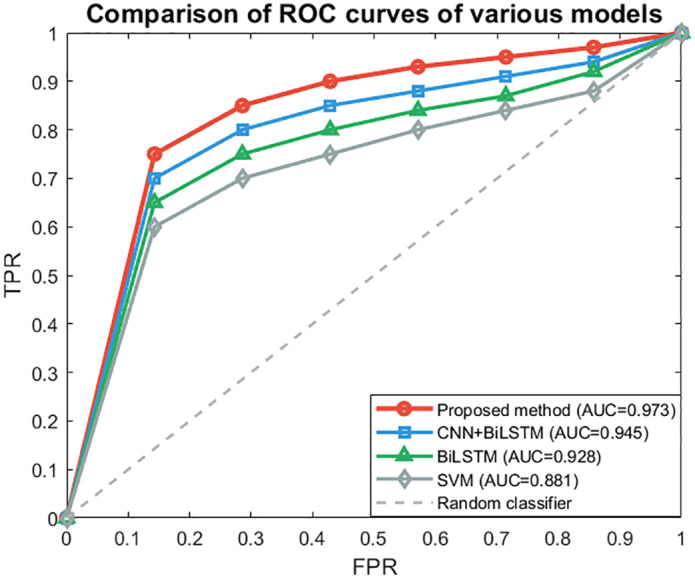
ROC curve.

From the model performance comparison chart in Fig 9 and the evaluation results of different models in Table 2, it can be seen that the accuracy, precision, recall, F1 and AUC of this model are significantly better than those of other models, and the accuracy is 3.7% higher than CNN bilstm without attention mechanism, indicating that attention mechanism can effectively improve the fusion effect. It is 4.9% higher than a single bilstm, reflecting the advantages of multi-source feature fusion. In the ROC curve in Fig 10, the ROC curve of the model in this paper is the closest to the upper left corner, and the AUC value is the highest, indicating that the method in this paper has the best comprehensive recognition ability under different thresholds.

**Table 2:** Performance comparison of different models.

Model name	Accuracy	Precision	Recall	F1	AU
SVM	85.2%	84.6%	83.1%	83.7%	88.1%
RF	87.4%	86.8%	84.9%	85.6%	89.7%
BiLSTM	91.3%	90.7%	88.6%	89.5%	92.8%
2D-CNN	90.7%	89.9%	87.5%	88.6%	91.9%
CNN + BiLSTM	92.5%	92.8%	91.7%	91.2%	94.5%
The proposed method	96.2%	95.8%	94.6%	95.1%	97.3%

The ablation experimental results in Table 3 clearly show the impact of different feature combinations and attention mechanisms on the performance of the model, highlighting the key role of multi-source feature fusion and attention mechanisms. From the perspective of single feature performance, the accuracy rate of the model with only DGA feature input is 88.4%, and the value of F1 is 86.7%, which is better than only current feature and only voiceprint feature, indicating that gas feature has basic recognition value in transformer condition assessment, but the information limitation of single feature leads to the overall low performance. After the fusion of double features, the performance is improved: the accuracy of DGA and current fusion is 91.7%, and value of F1 is 90.1%, which is significantly higher than other double feature combinations, reflecting the complementarity of gas features and electrical features. The fusion effect of DGA and voiceprint, current and voiceprint decreases in turn, indicating that the synergy of different features is different. When the three features were simply stitched and fused (without attention mechanism), the accuracy rate was improved to 92.5% and value of F1 was 91.2%, which further verified the necessity of multi-source information integration. After the introduction of attention mechanism, the accuracy and value of F1 of the complete model reached 96.2% and 95.1% respectively, which was about 3.7% higher than that of the three feature fusion without attention mechanism. It proves that attention mechanism can effectively focus on key features, suppress redundant information, and greatly enhance the fusion effect.

**Table 3:** Ablation experiment results.

Model configuration	Accuracy	F1	Explains
DGA features only	88.4%	86.7%	Single gas characteristic input
Current characteristics only	85.6%	83.2%	Single electrical feature input
Voiceprint signal only	82.3%	79.8%	Single vibration characteristic input
DGA+current	91.7%	90.1%	Dual feature fusion
DGA+voiceprint	89.2%	87.5%	Dual feature fusion
Current+voiceprint	87.9%	85.6%	Dual feature fusion
Three characteristics without attention	92.5%	91.2%	Simple splicing fusion
Three characteristics+attention	96.2%	95.1%	Full model configuration

The classification assessment results of each state category in Table 4 show that the proposed multi-source deep fusion state assessment model has excellent and balanced recognition performance for various operation states of transformers. Among them, the recognition effect of normal operation state (C_1_) is the best, which is closely related to the stability of equipment parameter characteristics under normal state and easy to be captured by the model. The recognition performance of winding overheating (C_2_) and iron core abnormality (C_4_) is also outstanding, with F1 score of 95.4% and 94.5% respectively. The former has a low rate of missed detection, while the latter has a strong judgment accuracy, which reflects the model’s effective ability to capture the characteristics of current fluctuations, voiceprint high-frequency anomalies and so on. The F1 scores of insulation deterioration (C_3_) and cooling system fault (C_5_) are relatively low, 92.0% and 90.7% respectively, which are related to the fact that the characteristics of insulation deterioration are easily disturbed, the proportion of cooling system fault samples is low and the characteristics are easy to be confused. From the macro average index, precision, recall and F1 score were 94.5%, 93.7% and 94.1% respectively. This further verifies the balance of the proposed model in the assessment of various state recognition, and fully illustrates the significant role of multi-source feature fusion and attention mechanism in improving the comprehensive discrimination ability of the model..

**Table 4:** Assessment results of each state category.

Status category	Precision	Recall	F1-score
C_1_	98.1%	97.3%	97.7%
C_2_	94.6%	96.2%	95.4%
C_3_	92.3%	91.8%	92.0%
C_4_	95.7%	93.4%	94.5%
C_5_	91.8%	89.7%	90.7%
Macro average	94.5%	93.7%	94.1%

## 6 Conclusions

This article explores in depth the online monitoring of transformer status and the fusion of multi-source data for the assessment of ultra-high voltage transformer status, and proposes a multi-source deep fusion state assessment model and method. This method combines 2D-CNN and BiLSTM network structures to construct a deep extraction and fusion discrimination mechanism for image and temporal features, and introduces attention mechanism and various comparative experiments to comprehensively verify the assessment accuracy and generalization ability of transformer operation status. Relevant case analysis was conducted on the monitoring data of the 1000kV ultra-high voltage main transformer at Dongwu Substation. The research results indicate that:

1) The method proposed in this article can effectively achieve accurate assessment of the state of ultra-high voltage transformers under complex operating conditions. Compared with traditional machine learning algorithms and single deep learning models, this model integrates multi-source features and attention mechanisms, which is more in line with the multidimensional characteristics of transformer state monitoring. Through comprehensive indicators such as macro average F1, it achieves comprehensive assessment from the perspective of classification balance and provides more reliable and detailed state discrimination results.
2) By quantifying the impact of different feature combinations on model performance, the key role of multi-source fusion and attention mechanism can be clearly reflected. When three feature fusion and attention mechanism are introduced, the model accuracy reaches 96.2%, which is 3.7% higher than the three feature fusion without attention mechanism. In addition, the performance gap between the AUC value of this model and the comparison model validates its ability to distinguish complex states and capture temporal features, providing a new multi-source fusion perspective for the state assessment of ultra-high voltage transformers and helping to systematically grasp the evolution laws of equipment operation status.

Deploying the multi-source feature extraction and fusion method proposed in the article in the actual transformer monitoring environment requires renovation of existing infrastructure and corresponding investment. Firstly, it is necessary to equip a multi-channel sensor platform to collect key variable data and ensure that the sensors match the operating characteristics of the transformer. In addition, in order to support the computing needs of the deep learning model, high-performance computing resources are also needed, which may involve edge computing or cloud computing platforms. After system deployment, continuous monitoring and maintenance of sensors and data processing modules are required to cope with dynamic changes in transformer operation status and equipment failures. In terms of investment, in addition to hardware infrastructure, it is also necessary to improve data transmission and processing capabilities, which may involve the integration and optimization of existing monitoring systems. In order to ensure the operability and continuous operation of the method, professional training and technical support for operators are also necessary.

In summary, although the method proposed in this article has significant theoretical advantages, its practical application still requires necessary investment in hardware upgrades, data processing platform construction, maintenance, and personnel training to ensure its effectiveness and sustainability in transformer monitoring environments.

## Supporting information

S1 Data Data set. (XLSX)
